# Comprehensive effects of functional agents on growth, nutrient accumulation, and rhizosphere bacterial communities in flue-cured tobacco (*Nicotiana tabacum* L.)

**DOI:** 10.3389/fpls.2026.1840341

**Published:** 2026-05-20

**Authors:** Minggang Chen, Yuanhuan Li, Ming Li, Guang Zhong, Dehu Xiang, Cheng Qu, Chongwen Zhu, Pengfei Yi, Lili Yang

**Affiliations:** 1 Changde Municipal Company, Hunan Provincial Tobacco Corporation, Changde, China; 2 College of Botanical Science and Technology, Hunan Biological and Electromechanical Polytechnic, Changsha, China

**Keywords:** carbon and nitrogen metabolism, dry matter accumulation, flue-cured tobacco, functional agents, rhizosphere bacterial community, source-sink allocation

## Abstract

**Introduction:**

Sustained nutrient supply and dry matter accumulation during the late field growth stage are crucial for the final yield and quality of flue-cured tobacco. However, the slow nutrient release of conventional organic fertilizers often restricts plant growth during this critical period.

**Methods:**

This study evaluated the comprehensive effects of supplementing conventional organic fertilizer with a carbon polymer water-soluble fertilizer alone (T2), or in further combination with an anti-continuous cropping agent (T3) and a microbial inoculant (T4).

**Results:**

The results demonstrated that, compared to the sole application of organic fertilizer, both the T3 and T4 combined treatments effectively promoted late-stage growth. Specifically, the T4 and T3 treatments significantly increased leaf dry matter accumulation by 39.84% and 29.62%, respectively. Regarding nutrient uptake, the T4 treatment significantly enhanced whole-plant nitrogen and phosphorus accumulation by 41.76% and 154.28%, respectively. Meanwhile, the T3 treatment significantly boosted whole-plant and leaf potassium accumulation by 62.43% and 124.59%, respectively. Crucially, multivariate analysis confirmed that the T4 treatment maximized dry matter by alleviating soil compaction (reducing bulk density), increasing soil available nutrients, and significantly activating glutamine synthetase and invertase in the leaves while maintaining the stable abundance of core dominant bacterial taxa. In contrast, the T3 treatment significantly altered soil acid-base conditions and selectively enriched specific taxa, including *Tepidisphaera* and *Gp16*, thereby improving the root zone microenvironment to facilitate a unique source-sink nutrient allocation.

**Discussion:**

In conclusion, supplementing the organic and carbon polymer fertilizer base with either an anti-continuous cropping agent or a microbial inoculant effectively overcomes late-stage growth bottlenecks. Specifically, the microbial inoculant combination (T4) demonstrated the optimal overall performance in maximizing dry matter accumulation and nitrogen/phosphorus uptake, while the anti-continuous cropping agent combination (T3) was optimal for enhancing leaf potassium accumulation. These combined applications achieve this by ameliorating soil physicochemical properties, improving the rhizosphere microenvironment, promoting carbon and nitrogen metabolism, and optimizing source-sink nutrient allocation. Ultimately, these findings provide practical fertilization strategies to alleviate late-stage premature senescence and optimize field nutrient management in flue-cured tobacco.

## 1 Introduction

Flue-cured tobacco (*Nicotiana tabacum* L.) is an important cash crop (Eickholt and Lewis, 2014). Its yield and economic benefits closely depend on cultivation and management measures during the field stage (Yang et al., 2024a). The field growth period is a critical stage for tobacco development. In particular, growth vigor and dry matter accumulation during the late growth stage directly determine the final yield and industrial quality of tobacco leaves (Lin et al., 2020; Liu et al., 2024). In recent years, tobacco production has widely adopted the increased application of organic fertilizers. This practice helps improve the physicochemical properties of tobacco-growing soil and mitigates microecological imbalances caused by excessive chemical fertilizer use (Bonanomi et al., 2020; Jiang et al., 2022). However, conventional organic fertilizers release nutrients relatively slowly. This slow release often fails to precisely match the intense demand for quick-acting nutrients during the rapid growth stage. Consequently, plants are highly susceptible to nutrient deficiency, premature senescence, or nutritional imbalance during the crucial late yield-forming stage. This ultimately limits further improvements in tobacco leaf quality (Guo et al., 2023).

Functional agents are agricultural inputs that effectively enhance fertilizer efficiency and improve the rhizosphere environment. Their primary growth-promoting pathways include providing quick-acting nutrients, activating soil nutrients, improving microecology, and enhancing plant stress resistance (Jalal et al., 2023; Ying et al., 2024). Different functional agents exhibit distinct modes of action and physiological effects. Carbon polymer organic water-soluble fertilizer is a polymeric organic water-soluble fertilizer that utilizes small-molecule carbon as a carrier. Roots can rapidly absorb and utilize these sources, providing direct energy for early plant growth and soil microorganisms (Wang et al., 2023). Anti-continuous cropping agents can improve the soil physicochemical and microbiological environment by degrading autotoxic substances or inhibiting soil-borne diseases. This process maintains late-stage root vitality and delays premature senescence (Trivedi et al., 2021). Meanwhile, microbial inoculants can colonize the rhizosphere and secrete phytohormones or extracellular enzymes (Dutta et al., 2022). These actions significantly activate insoluble phosphorus and potassium resources in the soil. Furthermore, they regulate plant endogenous hormone levels and carbon-nitrogen metabolism (Ahmad et al., 2018; Ouf et al., 2023). Beyond these direct physiological effects, these functional agents specifically alter the composition of the rhizosphere microbial community. This alteration is crucial for soil nutrient cycling (Malgioglio et al., 2022).

Existing research confirms the growth-promoting benefits of single functional agents. However, most studies remain limited to greenhouse experiments or the evaluation of a single product. These studies fail to fully capture the comprehensive fertilizer efficiency in complex field environments. This limitation is particularly evident in actual tobacco production. The relative fertilizer efficiency of combining organic fertilizers with different functional agents remains unknown. Furthermore, the comprehensive impact of these combinations on key physiological processes during the late growth stage is still unclear. To fill this gap, we investigated the practical application effects of supplementing conventional organic fertilizers with a carbon polymer water-soluble fertilizer alone, or in further cumulative combination with an anti-continuous cropping agent or a microbial inoculant under field conditions. Therefore, the specific objectives of this study are to compare the differences in the effects of various functional agents on the growth, nutrient accumulation, and source-sink allocation of flue-cured tobacco, to select the most suitable targeted fertilization strategy to improve the quality and efficiency of flue-cured tobacco in this region, and to elucidate the underlying mechanisms of action by characterizing the responses of leaf carbon-nitrogen metabolism and rhizosphere bacterial communities.

## 2 Materials and methods

### 2.1 Experimental site

The experiment was conducted in Nanbei Town, Shimen County, Changde City, Hunan Province (110°32′42″E, 29°52′14″N). The experimental site is located in a region with a subtropical humid monsoon climate, featuring an annual average temperature of 13.5 °C and an annual average precipitation of 1650 mm. The basic soil physicochemical properties were as follows: pH 6.50, soil bulk density (BD) 1.18 g/cm³, soil organic matter (SOM) 27.06 g/kg, total nitrogen (TN) 1.39 g/kg, alkali-hydrolyzable nitrogen (AN) 179.13 mg/kg, available phosphorus (AP) 19.56 mg/kg, and available potassium (AK) 379.88 mg/kg.

### 2.2 Fertilizer types

The fertilizers used in this study primarily included: tobacco-specific basal fertilizer (N:P_2_O_5_:K_2_O = 8:15:7, Hunan Jinye Zhongwang Technology Co., Ltd.); straw and rapeseed cake organic fertilizer (N + P_2_O_5_ + K_2_O ≥ 5%, organic matter ≥ 30%, Hunan Biye Agricultural Technology Development Co., Ltd.); carbon polymer organic water-soluble fertilizer (organic matter ≥ 100 g/kg, Tianjin Saimeile Import & Export Co., Ltd.); anti-continuous cropping agent primarily consists of *Actinomycetes* (viable count ≥ 5 × 10^9^ CFU/g, Beijing England Environmental Technology Co., Ltd.); microbial inoculant primarily consists of *Bacillus amyloliquefaciens* and *Bacillus subtilis* (viable count ≥ 6 × 10^10^ CFU/g, Tianjin Saimeile Import & Export Co., Ltd.); seedling-promoting fertilizer (N: P_2_O_5_:K_2_O = 20:9:0, Hunan Jinye Zhongwang Technology Co., Ltd.); Jinye Zhongwang compound fertilizer (N:P_2_O_5_:K_2_O = 10:5:29, Hunan Jinye Zhongwang Technology Co., Ltd.); and potassium sulfate (N:P_2_O_5_:K_2_O = 0:0:52, SDIC Xinjiang Luobupo Potash Co., Ltd.).

### 2.3 Field trials and design description

Field trials were conducted using the tobacco cultivar ‘Yun 87’. Before transplanting, the field was prepared for tobacco cultivation by raising ridges and applying basal fertilizer. Tobacco seedlings at the 8-leaf stage were selected and transplanted on ridges (in rings) with a plant-to-row spacing of 50 × 120 cm. Four treatments were established: T1, organic fertilizer (750 kg/ha); T2, organic fertilizer (750 kg/ha) + carbon polymer organic water-soluble fertilizer (60 kg/ha); T3, organic fertilizer (750 kg/ha) + carbon polymer organic water-soluble fertilizer (60 kg/ha) + anti-continuous cropping agent (30 kg/ha); and T4, organic fertilizer (750 kg/ha) + carbon polymer organic water-soluble fertilizer (60 kg/ha) + microbial inoculum (30 kg/ha). The application rates and methods of other standard fertilizers were consistent across all treatments. Specifically, the tobacco-specific basal fertilizer, organic fertilizer, carbon polymer organic water-soluble fertilizer, anti-continuous cropping agent, microbial inoculum, and potassium sulfate were thoroughly mixed and applied in bands as basal fertilizers. On the day of transplanting and at 7 days post-transplanting, the seedling-promoting fertilizer was applied with water. At 30 days post-transplanting, the compound fertilizer and potassium sulfate were applied as an aqueous solution via hole application, followed immediately by intertillage and hilling. Detailed fertilization schemes are presented in **Table 1**. The experiment was carried out under a randomized complete block design and repeated thrice with 12 plots (plot size = 6 m × 11 m per replication; 3 plots per treatment).

**Table 1:** Application methods of different treatments.

Fertilization time	Type of fertilizer	T1	T2	T3	T4
10 days before transplanting	Tobacco-specific basal fertilizer (kg/ha)	900	900	900	900
	Organic fertilizer (kg/ha)	750	750	750	750
	Carbon polymer organic water-soluble fertilizer (kg/ha)	0	60	60	60
	Anti-continuous cropping agent (kg/ha)	30	0	30	0
	Microbial inoculum (kg/ha)	0	30	0	30
	Potassium sulfate (kg/ha)	75	75	75	75
Day of transplanting	Seedling-promoting fertilizer (kg/ha)	37.5	37.5	37.5	37.5
7 days after transplanting	Seedling-promoting fertilizer (kg/ha)	37.5	37.5	37.5	37.5
30 days after transplanting	Compound fertilizer (kg/ha)	300	300	300	300
	Potassium sulfate (kg/ha)	300	300	300	300

### 2.4 Determination of agronomic traits

The agronomic traits of tobacco plants, including plant height (cm), stem circumference (cm), number of effective leaves, and maximum leaf length (cm) from each treatment after 30, 60 and 90 days of transplanting, were recorded according to the “Tobacco Industry Standard YC/T 142–1988 Tobacco Agronomic Trait Survey Methods” in China. Briefly, data were collected from 5 plants from each plot per treatment. The average value of these 5 plants was calculated to represent a single biological replicate for that plot, resulting in three true biological replicates (n=3) per treatment. The leaf area (cm^2^) was calculated using the following formula: leaf length × leaf width × 0.6345.

### 2.5 Determination of the accumulation of dry matter, total nitrogen, total phosphorus, and total potassium contents of tobacco plants

The accumulation of dry matter contents (g/plant) in different parts (root, stem, and leaf) of tobacco plants was determined at 30, 60 and 90 days of post-transplanting under various treatments. Briefly, 5 plants were randomly uprooted per plot from each treatment. These 5 plants were pooled to represent a single biological replicate for that plot (resulting in n=3 per treatment), and then divided into three parts (root, stem, and leaf). The collected samples were dry at 105 °C for 30 min, dried to constant weight at 80 °C for 48 h, and the contents of dry matter were measured in each part. The contents (mg/plant) of N, phosphorus (P), and potassium (K) were determined in different parts (root, stem, and leaf) of tobacco plants from the samples collected after 90 days of transplanting. The samples were digested with H_2_SO_4_-H_2_O_2_, and the contents of N, P, and K were determined with continuous flow analyzer, molybdenum-antimony anti-colorimetric, and flame photometric methods, respectively. Total dry matter accumulation and total nutrient accumulation of the plant were equal to the sum of roots, stems, and leaves (Yang et al., 2024b).

### 2.6 Determination of key carbon and nitrogen metabolism enzymes and metabolites

At 90 days post-transplanting, three representative tobacco plants were selected from each treatment. Fresh leaf samples were collected from the middle leaves (the 10th to 12th leaf positions) of each plant and pooled. For enzyme activity assays, the composite samples were wrapped in aluminum foil and gauze, immediately snap-frozen in liquid nitrogen for transport, and stored at −80 °C. Additional fresh leaf samples were oven-dried for the determination of carbon and nitrogen metabolites, including starch and reducing sugars. Nitrate reductase activity was determined using the *in vivo* method, and glutamine synthetase activity was measured via spectrophotometry (Gao et al., 2015). Invertase and amylase activities were assayed using the 3, 5-dinitrosalicylic acid colorimetric method (Li et al., 2017; Lei et al., 2022). The contents of starch and reducing sugars were quantified using a continuous flow analyzer (Zhang et al., 2024).

### 2.7 Sampling and determination of rhizosphere soil samples

Rhizosphere soil samples were collected 90 days after transplanting using the five-point sampling method. In each plot, soil from five plants was mixed thoroughly to form one composite sample. This sample was divided into two parts: one was stored at -80 °C for microbial analysis, and the other was air-dried for the analysis of soil properties.

For DNA extraction, 0.5 g of fresh soil was processed using the UltraClean Microbial DNA Isolation Kit (Mo Bio, USA). The V3–V4 region of the bacterial 16S rRNA gene was amplified using primers 338F (5′-ACTCCTACGGGAGGCAGCA-3′) and 806R (5′-GGACTACHVGGGTWTCTAAT-3′). The PCR reaction (25 μL) contained 12.5 μL 2× Premix Taq (TaKaRa), 1 μL of each primer (10 μM), 1 μL template DNA, and 9.5 μL distilled water. The PCR program was set as follows: 95 °C for 5 min; 35 cycles of 95 °C for 30 s, 55 °C for 30 s, and 72 °C for 45 s; and a final extension at 72 °C for 7 min. The PCR products were checked on a 1% agarose gel, purified, and sequenced on the Illumina HiSeq platform. Raw sequencing reads were assigned to each sample based on unique barcodes and trimmed to remove barcode and primer sequences. Paired-end reads were merged using FLASH (v1.2.11). Quality filtering of the raw reads was conducted using fqtrim (v0.94) to obtain high-quality clean reads. Chimeric sequences were detected and removed using Vsearch (v2.3.4). The high-quality sequences were then clustered into Operational Taxonomic Units (OTUs) at a 97% similarity threshold. Taxonomic classification of the representative bacterial sequences was assigned based on the SILVA database (release 132).

Standard methods were adopted to assess soil physicochemical attributes. BD was determined using the cutting ring method (Bao, 2000). For chemical properties, 10.0 g of air-dried soil was used to measure soil pH in a soil-water suspension (1:2.5, w/v) using a digital pH meter (Bao, 2000). SOM was quantified from 0.5 g of soil via the potassium dichromate oxidation method with external heating (Wang et al., 2025b). TN was measured using the Kjeldahl digestion method (Wang et al., 2025b). Available nutrients were determined as follows: AN was assayed using the alkali hydrolysis diffusion method with 2.0 g of soil; AP was extracted from 2.5 g of soil using 0.5 mol/L NaHCO_3_ and determined colorimetrically using the molybdenum blue method; and AK was extracted from 5.0 g of soil using 1 mol/L NH_4_OAc and measured by flame photometry (Wang et al., 2025b).

### 2.8 Statistical analysis

Data were initially organized using Microsoft Excel. Statistical analyses were performed using R software (version 4.5.3). One-way analysis of variance (ANOVA) followed by the least significant difference (LSD) test was used to determine significant differences among treatments at *p* < 0.05. Pearson correlation analysis was also conducted using R software. Redundancy analysis (RDA) was performed using the Chiplot online platform (https://www.chiplot.online/, accessed on 29 April 2026). For microbial community analysis, α-diversity was evaluated using the Shannon, Chao1, ACE, and Simpson indices. β-diversity was assessed via Non-metric Multidimensional Scaling (NMDS). Stacked bar plots were employed to visualize the taxonomic composition and variations in species abundance at the genus level.

## 3 Results

### 3.1 Effects of combined application of organic fertilizer and different functional agents on agronomic trait

The results demonstrated that at 30 days after transplantation, all agronomic trait indices showed no significant differences among the fertilization treatments (**Table 2**). At 60 days of post-transplantation, the stem girth under treatment T4 was significantly higher than that under treatments T1, T2, and T3. Meanwhile, the maximum leaf width and leaf area under treatments T3 and T4 were significantly higher than those under T2. Other indices showed no significant differences among all treatments during this period. However, after 90 days of transplantation, the plant height under treatment T3 was significantly higher than that under T1 (*p* < 0.05). In addition, the maximum leaf width and the number of leaves under treatment T4 were significantly higher than those under T2 and T3. These indicates that the combined application of an anti-continuous cropping agent (T3) or a microbial inoculum (T4) promotes the middle and late-stage growth of flue-cured tobacco. Specifically, these treatments improve stem development, leaf expansion, and plant height. In contrast, the sole addition of the carbon polymer organic water-soluble fertilizer (T2) does not significantly improve these agronomic traits.

**Table 2:** Effects of different fertilization treatments on main agronomic traits of flue-cured tobacco at different growth stages.

Days after transplantation	Treatments	Plant height (cm)	Stem girth (cm)	Maximum leaf length (cm)	Maximum leaf width (cm)	Number of leaves	Leaf area (cm²)
30d	T1	26.53 ± 4.50a	4.07 ± 0.25a	48.40 ± 3.99a	28.10 ± 1.91a	13.33 ± 0.58a	861.27 ± 67.19a
	T2	30.50 ± 1.68a	4.63 ± 0.45a	48.03 ± 3.33a	27.53 ± 2.40a	12.67 ± 1.15a	836.71 ± 50.44a
	T3	25.93 ± 6.06a	4.93 ± 0.81a	48.77 ± 5.00a	29.23 ± 1.62a	13.67 ± 1.53a	902.40 ± 71.68a
	T4	25.50 ± 3.34a	4.97 ± 0.81a	49.70 ± 1.90a	28.70 ± 2.25a	12.33 ± 0.58a	904.55 ± 71.06a
60d	T1	92.60 ± 12.97a	9.73 ± 0.40b	71.63 ± 2.47a	34.33 ± 0.06ab	20.67 ± 0.58a	1560.44 ± 51.17ab
	T2	99.90 ± 9.83a	9.67 ± 0.15b	72.27 ± 1.50a	31.67 ± 1.14b	21.00 ± 1.00a	1452.74 ± 81.81b
	T3	104.03 ± 3.55a	10.00 ± 0.26b	76.00 ± 4.92a	37.50 ± 2.70a	21.33 ± 0.58a	1811.89 ± 221.04a
	T4	103.30 ± 6.22a	10.93 ± 0.75a	75.17 ± 1.60a	36.83 ± 2.35a	20.67 ± 0.58a	1757.81 ± 139.30a
90d	T1	103.83 ± 7.29b	10.77 ± 0.23a	84.10 ± 6.99a	40.50 ± 2.86ab	14.33 ± 0.58ab	2165.65 ± 286.35a
	T2	111.53 ± 2.72ab	11.00 ± 0.44a	79.87 ± 5.65a	37.43 ± 0.47b	13.33 ± 0.58b	1896.51 ± 127.75a
	T3	118.00 ± 5.22a	11.03 ± 0.55a	80.20 ± 4.76a	37.73 ± 1.63b	13.33 ± 0.58b	1922.95 ± 192.72a
	T4	112.67 ± 3.06ab	11.27 ± 0.21a	84.37 ± 6.03a	42.67 ± 2.81a	14.67 ± 0.58a	2282.68 ± 197.05a

Data are presented as mean ± standard deviation (n = 3). Different lowercase letters within the same column indicate significant differences among treatments at *p* < 0.05 according to the least significant difference (LSD) test.

### 3.2 Effects of combined application of organic fertilizer and different functional agents on dry matter accumulation and distribution ratio in flue-cured tobacco

Different fertilization treatments significantly affected total dry matter accumulation and its distribution ratio among organs of flue-cured tobacco at various growth stages, showing clear stage-specific characteristics (**Table 3**). At 30 days after transplantation, T2 and T4 effectively promoted early dry matter accumulation. Total dry matter accumulation of the whole plant under T2 and T4 was significantly higher than under T1 (*p* < 0.05). Leaf dry matter accumulation showed no significant differences among treatments during this stage. The increase in total accumulation mainly resulted from root and stem development, with root dry matter under T2 significantly higher than T1 and T3, and stem dry matter under T4 being significantly the highest. At 60 days after transplantation, dry matter accumulation exhibited different trends. Total dry matter accumulation under T1 and T2 was significantly higher than T3 and T4. Regarding the dry matter distribution pattern, T2, T3, and T4 all began showing a trend of optimizing dry matter transfer to leaves, where total leaf dry matter accumulation and leaf distribution ratio under T2 reached the highest levels, significantly outperforming T1. At 90 days after transplantation, T3 and T4 demonstrated obvious late-stage growth advantages and material synthesis capabilities. Total dry matter accumulation under T4 reached the highest level, significantly exceeding all other treatments, followed by T3, which was also significantly higher than T1 and T2. For the core economic organs, leaf dry matter accumulation under T4 and T3 significantly increased by 39.84% and 29.62%, respectively, compared to T1. Based on the final material distribution ratios, leaf dry matter distribution ratios under T3 and T4 were significantly higher than T1 and T2, with stem distribution ratios significantly reduced accordingly.

**Table 3:** Effects of different fertilization treatments on dry matter accumulation and distribution of flue-cured tobacco at different growth stages.

Days after transplantation	Treatments	Dry matter accumulation (g/plant)	Dry matter accumulation (g/plant)			Dry matter distribution ratio (%)		
			Root	Stem	Leaf	Root	Stem	Leaf
30d	T1	27.87 ± 3.29b	3.42 ± 0.32b	4.21 ± 0.89b	20.23 ± 2.14a	12.32 ± 0.86a	15.00 ± 1.35b	72.68 ± 1.05a
	T2	34.27 ± 2.02a	5.47 ± 1.29a	5.46 ± 1.19ab	23.34 ± 2.04a	16.08 ± 4.33a	15.84 ± 2.52b	68.08 ± 3.74b
	T3	32.37 ± 3.70ab	4.10 ± 0.26b	5.47 ± 1.41ab	22.79 ± 2.53a	12.82 ± 1.95a	16.72 ± 2.67ab	70.46 ± 1.77ab
	T4	36.18 ± 2.78a	4.70 ± 0.45ab	7.21 ± 0.44a	24.28 ± 2.43a	12.99 ± 0.90a	19.99 ± 1.94a	67.02 ± 1.58b
60d	T1	129.79 ± 8.68a	23.23 ± 1.23a	47.50 ± 3.05a	59.06 ± 6.07b	17.91 ± 0.53a	36.64 ± 1.80a	45.45 ± 2.31b
	T2	129.44 ± 7.33a	21.37 ± 0.89b	39.16 ± 3.62b	68.91 ± 4.66a	16.52 ± 0.31b	30.25 ± 1.99b	53.23 ± 1.84a
	T3	113.81 ± 2.86b	12.74 ± 0.45d	39.62 ± 2.46b	61.45 ± 4.60ab	11.20 ± 0.46d	34.84 ± 2.58a	53.97 ± 3.04a
	T4	111.65 ± 2.46b	14.97 ± 0.58c	39.48 ± 0.79b	57.20 ± 1.28b	13.41 ± 0.35c	35.36 ± 0.10a	51.23 ± 0.28a
90d	T1	237.28 ± 3.07c	58.32 ± 4.15b	54.10 ± 2.03b	124.86 ± 2.92c	24.58 ± 1.74a	22.80 ± 0.86ab	52.62 ± 0.91c
	T2	253.00 ± 6.00c	58.37 ± 4.15b	59.50 ± 6.30ab	135.14 ± 5.64c	23.10 ± 2.18a	23.49 ± 2.00a	53.40 ± 1.40bc
	T3	283.45 ± 8.48b	63.66 ± 7.03b	57.95 ± 4.77ab	161.84 ± 1.89b	22.43 ± 1.88a	20.45 ± 1.68b	57.12 ± 1.41a
	T4	314.55 ± 19.33a	74.72 ± 3.98a	65.23 ± 5.39a	174.60 ± 10.73a	23.76 ± 0.31a	20.72 ± 0.70b	55.51 ± 0.86ab

Data are presented as mean ± standard deviation (n = 3). Different lowercase letters within the same column indicate significant differences among treatments at *p* < 0.05 according to the least significant difference (LSD) test.

### 3.3 Effects of combined application of organic fertilizer and different functional agents on nitrogen accumulation and distribution in flue-cured tobacco

**Figure 1:**
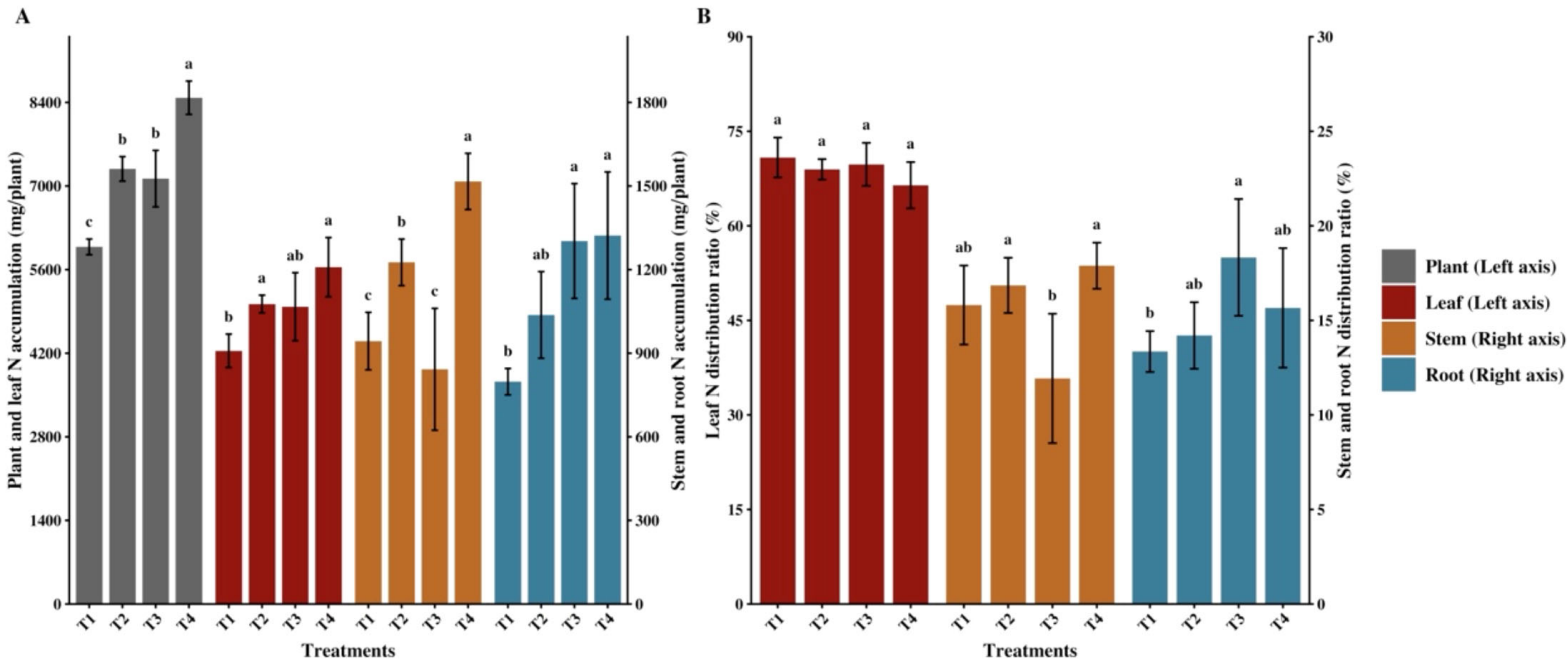
Effects of combined application of organic fertilizer and different functional agents on nitrogen (N) accumulation and distribution in flue-cured tobacco. **(A)** N accumulation in the whole plant, leaf, stem, and root across different treatments. **(B)** N distribution ratio in the leaf, stem, and root. Data are presented as mean ± standard deviation (n = 3). Different lowercase letters indicate significant differences among treatments at *p* < 0.05 according to the least significant difference (LSD) test.

The combined application of organic fertilizer and different functional agents significantly affected N accumulation in flue-cured tobacco, while their effects on N distribution ratios were relatively minor. Compared with the control (T1), all combined application treatments (T2, T3, and T4) significantly increased N accumulation in the whole plant and leaves (*p* < 0.05) (**Figure 1A**). Specifically, N accumulation in the whole plant and leaves under T4 reached the highest levels, significantly increasing by 41.76% and 33.02% compared to T1, respectively, followed by T2 and T3. Regarding root and stem N accumulation, only root N accumulation under T3 was significantly higher than T1, whereas other treatments showed no significant differences. N distribution analysis (**Figure 1B**) revealed that N was primarily distributed in leaves (66.44%–70.84%) across all treatments, and neither leaf nor stem N distribution ratios under T2, T3, and T4 differed significantly from T1. Only T3 specifically altered the distribution pattern, exhibiting a significantly higher root N distribution ratio than T1.

### 3.4 Effects of combined application of organic fertilizer and different functional agents on phosphorus accumulation and distribution in flue-cured tobacco

**Figure 2:**
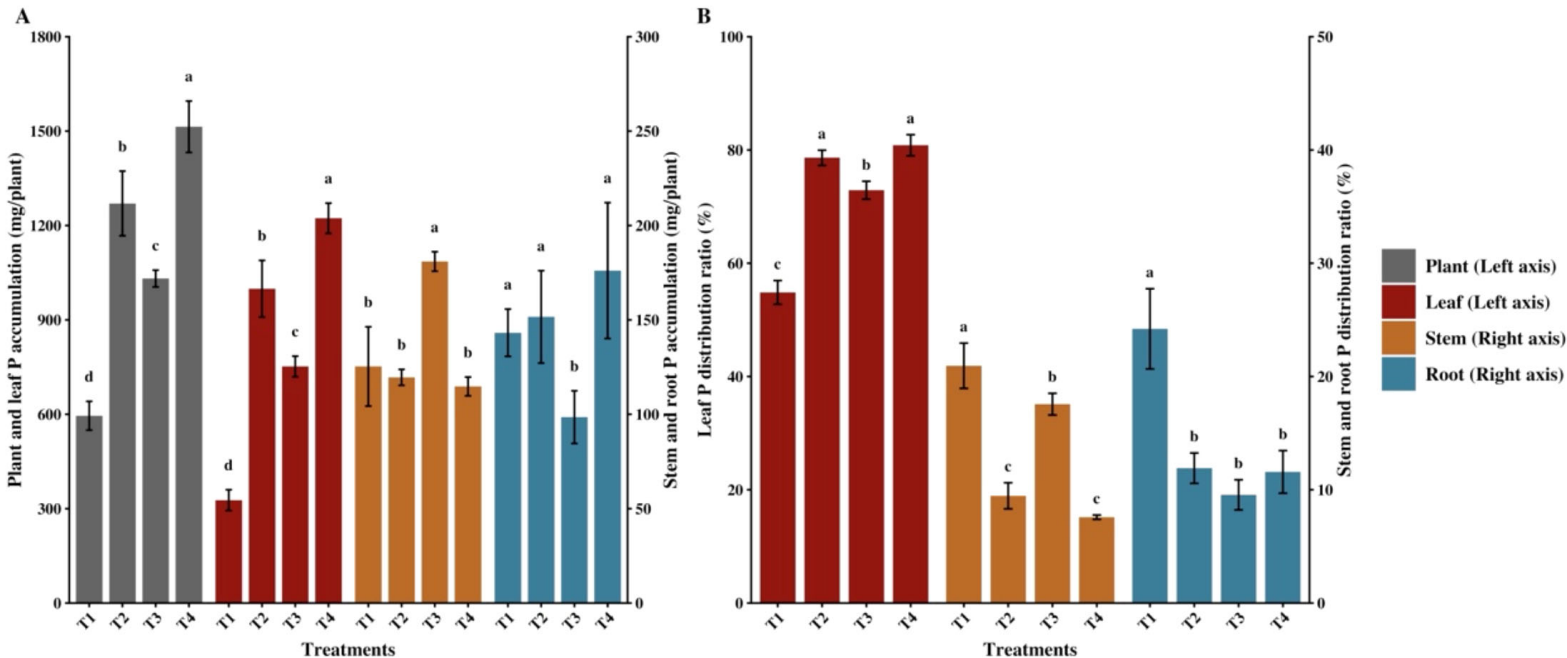
Effects of combined application of organic fertilizer and different functional agents on phosphorus (P) accumulation and distribution in flue-cured tobacco. **(A)** P accumulation in the whole plant, leaf, stem, and root across different treatments. **(B)** P distribution ratio in the leaf, stem, and root. Data are presented as mean ± standard deviation (n = 3). Different lowercase letters indicate significant differences among treatments at *p* < 0.05 according to the least significant difference (LSD) test.

The combined application of organic fertilizer and different functional agents significantly affected P accumulation and distribution patterns in flue-cured tobacco (**Figure 2A**). Compared with the control (T1), all combined application treatments (T2, T3, and T4) significantly increased P accumulation in the whole plant and leaves (*p* < 0.05). Specifically, P accumulation in the whole plant and leaves under T4 reached the highest levels, significantly increasing by 154.28% and 274.17% compared to T1, respectively, followed by T2 and T3, which were also significantly higher than T1. Regarding stem and root P accumulation, only T3 showed significantly higher stem accumulation and lower root accumulation than T1, whereas other treatments showed no significant differences from T1. P distribution analysis revealed that all combined application treatments significantly optimized P distribution within the plant (**Figure 2B**). Compared to T1, treatments T2, T3, and T4 significantly increased the leaf P distribution ratio, with T4 and T2 reaching the highest levels. Concurrently, the root and stem P distribution ratios under all combined application treatments were significantly lower than those under T1, indicating that the combined applications not only promoted P absorption but also strongly drove the directional transfer of P to core economic organs.

### 3.5 Effects of combined application of bio-organic fertilizer and different functional agents on potassium accumulation and distribution in flue-cured tobacco

**Figure 3:**
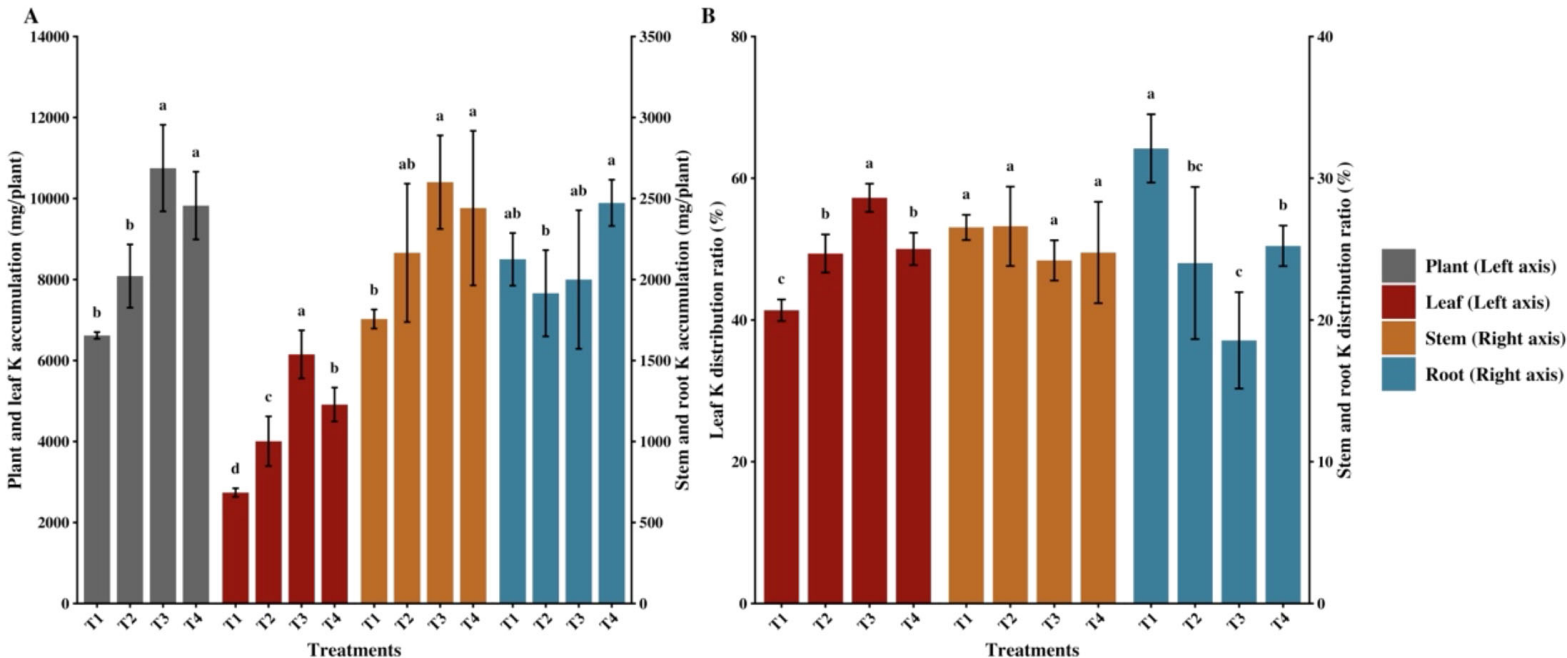
Effects of combined application of organic fertilizer and different functional agents on potassium (K) accumulation and distribution in flue-cured tobacco. **(A)** K accumulation in the whole plant, leaf, stem, and root across different treatments. **(B)** K distribution ratio in the leaf, stem, and root. Data are presented as mean ± standard deviation (n = 3). Different lowercase letters indicate significant differences among treatments at *p* < 0.05 according to the least significant difference (LSD) test.

The combined application of bio-organic fertilizer and different functional agents significantly affected K accumulation and distribution in flue-cured tobacco (**Figure 3A**). Compared with the control (T1), treatments T3 and T4 significantly increased total K accumulation in the whole plant (*p* < 0.05) by 62.43% and 48.45%, respectively, while T2 showed no significant difference from T1. For leaf K accumulation, all combined application treatments (T2, T3, and T4) were significantly higher than T1, with T3 reaching the highest level, representing a 124.59% increase over T1. Regarding stem accumulation, T3 and T4 were significantly higher than T1; however, root K accumulation showed no significant differences between any combined application treatments and T1. K distribution analysis (**Figure 3B**) indicated that all combined application treatments significantly promoted K transfer to leaves. Compared to T1, treatments T2, T3, and T4 significantly increased the leaf K distribution ratio, with T3 achieving the highest rate. Concurrently, the root K distribution ratios under all combined application treatments were significantly lower than T1, while stem K distribution ratios showed no significant differences among all treatments.

### 3.6 Effects of combined application of organic fertilizer and different functional agents on carbon and nitrogen metabolism in the late growth stage of flue-cured tobacco

The combined application of organic fertilizer and different functional agents had varying effects on key carbon and nitrogen metabolism enzyme activities and related product contents in the late growth stage of flue-cured tobacco (**Table 4**). Compared with the control (T1), treatment T4 significantly increased the activities of glutamine synthetase and invertase (*p* < 0.05), by 29.10% and 26.59%, respectively. However, nitrate reductase activity showed no significant differences among all treatments. Regarding α-amylase activity, only T3 was significantly lower than T1, whereas other treatments showed no significant differences from T1. For metabolites, T4 significantly increased the starch content in tobacco leaves by 16.08% compared to T1, while T2 and T3 showed no significant differences from T1. Furthermore, the reducing sugar contents under all combined application treatments (T2, T3, and T4) were not significantly different from the T1 control. Overall, the combined application of the microbial inoculum (T4) effectively enhanced the activities of key carbon and nitrogen metabolism enzymes and significantly promoted starch accumulation during the late growth stage.

**Table 4:** Effects of different treatments on key carbon and nitrogen metabolism enzyme activities and related product contents in the late growth stage of flue-cured tobacco.

Treatments	α-Amylase (mg/min/g)	Glutamine Synthetase (U/g)	Nitrate Reductase (U/g)	Invertase (U/g)	Reducing Sugar (%)	Starch (%)
T1	1.43 ± 0.12ab	6.46 ± 0.34b	0.22 ± 0.02a	65.28 ± 8.53b	1.00 ± 0.07ab	31.46 ± 0.31b
T2	1.28 ± 0.09bc	6.66 ± 0.37b	0.22 ± 0.03a	64.96 ± 2.09b	1.05 ± 0.03a	35.07 ± 0.71ab
T3	1.04 ± 0.06c	5.74 ± 0.33b	0.22 ± 0.02a	65.94 ± 5.73b	0.90 ± 0.06b	32.61 ± 1.45ab
T4	1.57 ± 0.21a	8.34 ± 1.08a	0.24 ± 0.02a	82.64 ± 3.35a	0.98 ± 0.05ab	36.52 ± 3.95a

Data are presented as mean ± standard deviation (n = 3). Different lowercase letters within the same column indicate significant differences among treatments at *p* < 0.05 according to the least significant difference (LSD) test.

### 3.7 Effects of combined application of organic fertilizer and different functional agents on physicochemical properties of tobacco-planting soil

The combined application of organic fertilizer and different functional agents significantly affected the physicochemical properties of tobacco-planting soil (**Table 5**). Compared with T1, the combined application treatments (T2, T3, and T4) all significantly increased soil nutrient contents and improved the soil environment (*p* < 0.05). Compared to T1, treatments T2, T3, and T4 significantly increased AN, AP, AK, and SOM by 24.64% to 50.20%, 18.52% to 39.29%, 19.30% to 47.39%, and 2.71% to 8.66%, respectively, with all indicators reaching their highest levels under the T4 treatment. Regarding soil acidity and physical structure, the T3 treatment showed the most prominent effect in increasing pH, which was significantly higher than T1 by 10.31%. Concurrently, the BD under all combined treatments was significantly lower than that of T1, with T4 showing the largest reduction of 4.13%. These results indicate that the combined application of functional agents not only effectively promoted the activation and accumulation of key soil nutrients but also markedly ameliorated soil acidification and compaction.

**Table 5:** Effects of combined application of organic fertilizer and different functional agents on physicochemical properties of tobacco-planting soil.

Treatments	pH	SOM (g/kg)	TN (g/kg)		BD (g/cm^3^)	AN (mg/kg)	AP (mg/kg)	AK (mg/kg)
T1	5.53 ± 0.14c	28.05 ± 0.75b	1.35 ± 0.01c	1.21 ± 0.02a		143.44 ± 6.51c	27.16 ± 1.08c	307.67 ± 4.59d
T2	5.73 ± 0.05bc	28.81 ± 1.08ab	1.39 ± 0.04bc	1.19 ± 0.02ab		178.79 ± 3.94b	32.19 ± 2.63b	367.06 ± 4.72c
T3	6.10 ± 0.12a	29.83 ± 0.72a	1.44 ± 0.06ab	1.17 ± 0.03ab		185.56 ± 3.69b	33.42 ± 1.34b	425.67 ± 3.01b
T4	5.80 ± 0.07b	30.48 ± 0.99a	1.47 ± 0.04a	1.16 ± 0.03b		215.44 ± 4.19a	37.83 ± 1.24a	453.46 ± 2.53a

Data are presented as mean ± standard deviation (n = 3). Different lowercase letters within the same column indicate significant differences among treatments at *p* < 0.05 according to the least significant difference (LSD) test.

### 3.8 Diversity and differential analysis of soil bacterial communities

Different treatments had varying effects on the α diversity of the soil bacterial community (**Table 6**). Regarding indices reflecting community species richness, compared with the control (T1), treatments T2 and T4 significantly increased the Chao1 and Ace indices of soil bacteria (*p* < 0.05), reaching the highest levels with no significant difference between them. Conversely, the Chao1 and Ace indices under T3 were significantly lower than those under T1. However, concerning the Shannon and Simpson indices, which reflect overall community diversity and evenness, there were no significant differences between any of the combined application treatments (T2, T3, and T4) and T1 (*p* > 0.05). Overall, these results indicate that the combined application of carbon polymer organic water-soluble fertilizer (T2) and microbial inoculum (T4) significantly increased soil bacterial species richness, whereas the anti-continuous cropping agent (T3) significantly decreased bacterial richness; nevertheless, none of these combined treatments fundamentally altered the overall diversity structure of the soil bacterial community.

**Table 6:** Analysis of the α diversity of bacteria.

Treatments	Shannon	Chao1	Ace	Simpson
T1	6.08 ± 0.25a	22036.73 ± 249.39b	22028.10 ± 214.92b	0.10 ± 0.00a
T2	6.35 ± 0.34a	23402.26 ± 206.09a	23355.95 ± 109.29a	0.11 ± 0.03a
T3	5.92 ± 0.26a	19889.75 ± 641.29c	19480.32 ± 612.51c	0.12 ± 0.03a
T4	6.34 ± 0.06a	23878.88 ± 1031.96a	23779.14 ± 1032.99a	0.12 ± 0.02a

Data are presented as mean ± standard deviation (n = 3). Different lowercase letters within the same column indicate significant differences among treatments at *p* < 0.05 according to the least significant difference (LSD) test.

**Figure 4:**
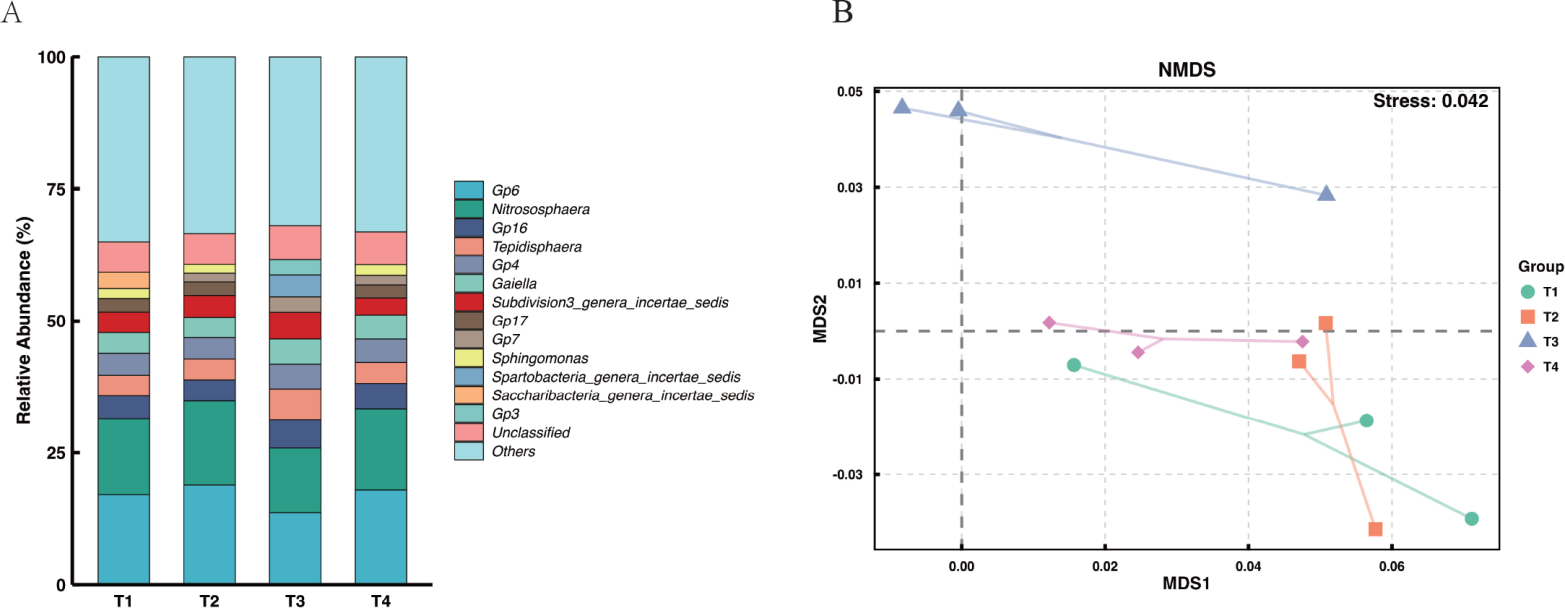
Impact of different fertilization treatments on the composition and diversity of the soil bacterial community. **(A)** Relative abundance of dominant bacterial taxa at the genus level across different treatments. **(B)** Non-metric Multidimensional Scaling (NMDS) analysis showing the beta-diversity of bacterial communities (Stress = 0.042).

Genus-level bacterial community composition analysis (**Figure 4A**) indicated that the community structures in treatments T2 and T4 were highly similar to the T1 control, with *Gp6* and *Nitrososphaera* remaining the absolute dominant genera (accounting for 17.09%–18.91% and 14.28%–15.84% of the total sequences, respectively). In contrast, the T3 treatment (combined application of an anti-continuous cropping agent) caused a distinct shift in community composition. Under this treatment, the relative abundances of the dominant resident taxa *Gp6* and *Nitrososphaera* decreased to 13.71% and 12.17%, respectively, while the proportions of specific functional taxa such as *Tepidisphaera* and *Gp16* significantly increased to 5.92% and 5.29%, respectively. This shift in species abundance suggests that the T3 treatment promoted the proliferation of adaptable microbial populations, partially replacing sensitive resident taxa.

Non-metric multidimensional scaling (NMDS) analysis (**Figure 4B**) confirmed these structural shifts. The scatter plot (Stress = 0.042) displayed distinct clustering patterns. Notably, the T3 samples formed a cluster clearly separated from T1, T2, and T4. This separation indicates that, compared with the T1 control, the T3 treatment significantly altered the overall structure of the soil bacterial community.

### 3.9 Multivariate analysis of soil, plant, and microbial factors

**Figure 5:**
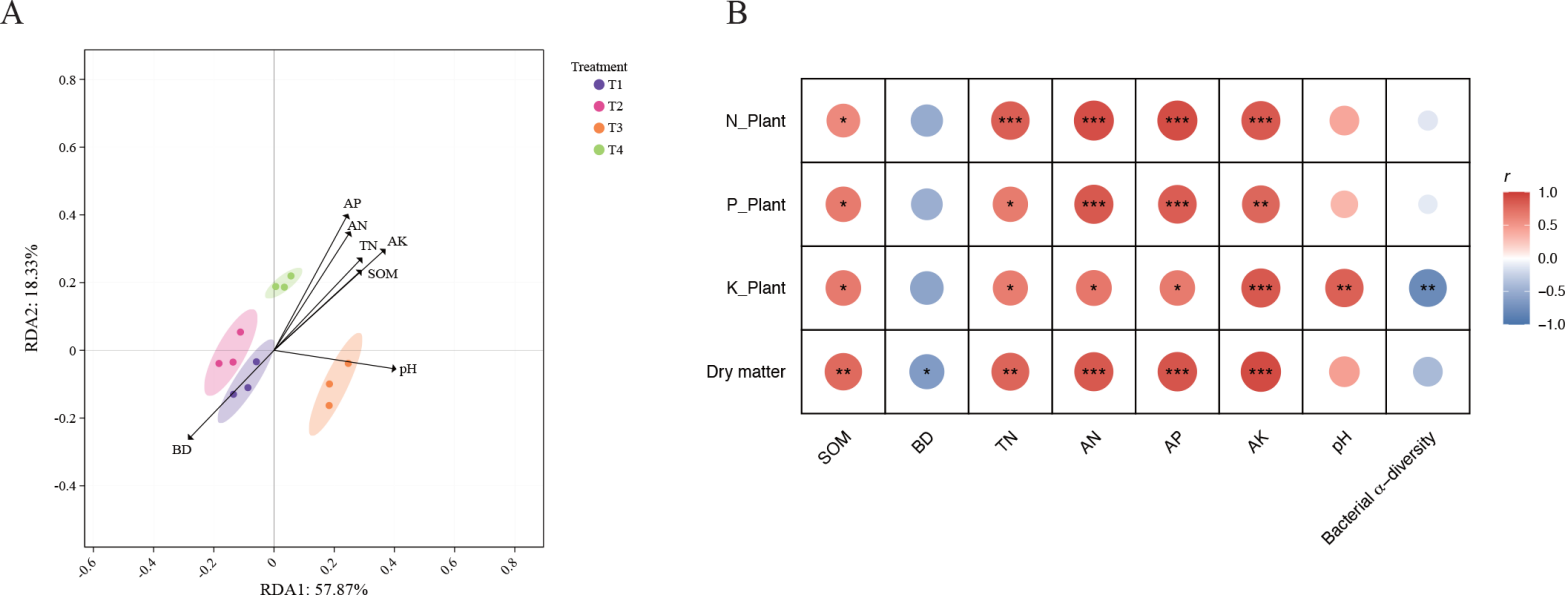
Relationships among soil physicochemical properties, tobacco growth, and bacterial diversity under different fertilization treatments. **(A)** Redundancy analysis (RDA) biplot illustrating the distribution of soil samples under different treatments constrained by soil physicochemical factors. **(B)** Pearson correlation heatmap evaluating the relationships among soil physicochemical properties, bacterial α-diversity, and plant growth metrics (N_Plant, P_Plant, K_Plant, and Dry matter). Red and blue circles indicate positive and negative correlations, respectively. Color intensity and circle size are proportional to the correlation coefficients (r). Asterisks denote statistical significance (*p < 0.05, **p < 0.01, ***p < 0.001). Abbreviations: SOM, soil organic matter; BD, bulk density; TN, total nitrogen; AN, available nitrogen; AP, available phosphorus; AK, available potassium.

Redundancy analysis (RDA) illustrates the differences in the soil microenvironment under various fertilizer combinations (**Figure 5A**), with the first two axes cumulatively explaining 76.2% of the variance. In the RDA biplot, the vectors representing SOM, TN, AN, AP, and AK are clustered together and point in the opposite direction to the BD vector. The sample distributions for the control (T1) and the application of carbon polymer alone (T2) align mainly with the BD vector, indicating relatively higher BD and a limited increase in available nutrients under these basal treatments. The distribution of the treatment with the anti-continuous cropping agent (T3) aligns primarily with the pH vector, showing that this treatment notably changed the soil acid-base conditions. In contrast, the sample points of the treatment with the microbial inoculum (T4) align with all core nutrient vectors, demonstrating that this treatment is more effective in increasing AN, AP, and AK, promoting SOM accumulation, and reducing BD, thereby providing a favorable physicochemical environment for tobacco growth.

Pearson correlation analysis demonstrates that soil physicochemical properties significantly affect tobacco growth and nutrient absorption (**Figure 5B**). The results show that the nitrogen, phosphorus, and potassium contents, as well as dry matter accumulation in tobacco plants, exhibit significant or highly significant positive correlations with SOM, TN, AN, AP, and AK. This confirms that increasing SOM and available nutrient contents plays a key role in promoting plant biomass accumulation. Conversely, BD shows a significant negative correlation with dry matter and multiple nutrient indices, indicating that soil compaction restricts tobacco growth and development. Furthermore, pH is highly significantly and positively correlated with plant potassium content, whereas bacterial $\alpha$-diversity shows a highly significant negative correlation. This indicates that under specific micro-ecological conditions, variations in bacterial community diversity may alter the microenvironment and consequently affect potassium bioavailability.

## 4 Discussion

Favorable agronomic traits and dry matter accumulation form the material basis for the yield of flue-cured tobacco. The results of this study show that the growth-promoting effects of different functional agents exhibit significant stage-specific characteristics. During the early transplanting stage, the combined application of the carbon polymer organic water-soluble fertilizer (T2) showed an initial growth-promoting advantage. This occurred because tobacco roots could rapidly absorb its quick-acting carbon sources and small-molecule nutrients (Wang et al., 2025a). However, relying solely on these quick-acting nutrients (T2) cannot sustain the vigorous demands of the plants during the late growth stage. Building upon this carbon polymer fertilizer base, the further addition of an anti-continuous cropping agent (T3) or a microbial inoculant (T4) effectively overcame this obstacle. During the late growth stage, both the T3 and T4 treatments significantly increased plant height and stem girth. Furthermore, their total dry matter accumulation was higher than that of the T2 treatment alone. This promoting effect is directly supported by our redundancy analysis (RDA) and Pearson correlation analysis. Specifically, the correlation analysis demonstrates a significant positive relationship between soil available nutrients, soil organic matter, and dry matter accumulation, alongside a significant negative correlation with bulk density. The RDA biplot confirms that the T4 treatment provides a favorable physicochemical environment by aligning with core nutrient vectors and opposing the bulk density vector. Concurrently, the microenvironmental shift driven by the T3 treatment aligns with the established ecological function of anti-continuous cropping agents in degrading autotoxic substances and inhibiting soil-borne pathogens, thereby fundamentally improving the soil physicochemical and microbiological environment (Wang et al., 2022). Meanwhile, the introduced microbial inoculant stimulated root development by secreting phytohormones, maintaining robust plant vitality during the late stage (Saeed et al., 2021). Furthermore, the “source-sink” allocation pattern of dry matter directly determines the final economic value of tobacco leaves (Andrews et al., 2005; Smith et al., 2018). In this study, the T3 and T4 treatments significantly increased total dry matter during the late growth stage. More importantly, they drove the directional transfer of photosynthates to the leaves, which significantly increased the dry matter distribution ratio in the leaves. During the late growth stage, tobacco leaves act as both the “source” for photosynthesis and the main “sink” for dry matter accumulation. This significant advantage in growth and material allocation inevitably requires stronger underlying nutrient supply and metabolic activities for support. This highly efficient dry matter accumulation is directly supported by the optimized soil microenvironment demonstrated in our multivariate analysis.

Highly efficient absorption and directional allocation of mineral nutrients serve as the physiological basis for the late-stage surge in dry matter and quality formation of flue-cured tobacco (Yu et al., 2026). Previous studies have shown that applying beneficial microorganisms and functional organic fertilizers can significantly activate insoluble soil nutrients by secreting organic acids or enzymes. This activation improves the absorption efficiency of nitrogen, phosphorus, and potassium in crops and optimizes their transfer to the aboveground parts (Ouf et al., 2023; Pavankumar et al., 2024). Our statistical analysis provides robust evidence for this mechanism: plant nutrient contents exhibit highly significant positive correlations with SOM and available nutrients. We found that the combined application of different functional agents (T2, T3, and T4) significantly increased the overall accumulation of nitrogen, phosphorus, and potassium during the late stage. These treatments also drove the directional transfer of phosphorus and potassium to the leaves. The substantial enrichment of phosphorus and potassium in the leaves is a prerequisite for maintaining high levels of photophosphorylation and energy metabolism. It is also key to improving the final combustibility of the tobacco leaves (Mohamed et al., 2021). However, different agents exhibited specific response mechanisms regarding nutrient allocation. Unlike the general trend of transferring all nutrients to the aboveground parts, the combined treatment containing the anti-continuous cropping agent (T3) showed a unique pattern. It promoted the directional transfer of phosphorus and potassium to the leaves to ensure late-stage photosynthetic efficiency. Additionally, it specifically increased the nitrogen distribution ratio in the roots to delay premature root senescence. The RDA results further elucidate this specific mechanism: the T3 treatment distinctly altered soil acid-base conditions, aligning with the pH vector, which is highly significantly and positively correlated with plant potassium content. This synergistic aboveground and underground nutrient allocation strategy effectively maintained the continuous transport of nutrients and water. Consequently, this strategy laid a solid material foundation for the highly efficient dry matter accumulation in the late stage.

Carbon and nitrogen metabolism is a core physiological process determining substance accumulation and the final quality of tobacco leaves. Previous studies indicate that microbial fertilizers can effectively regulate crop metabolism (Lai et al., 2024; Liang et al., 2025). Consistent with these findings, our study showed that the combined treatment incorporating the microbial inoculant (T4) significantly increased the activities of glutamine synthetase and invertase in leaves during the late growth stage. Consequently, this increase significantly boosted starch accumulation, suggesting that beneficial microbiota enhance nutrient uptake by optimizing the rhizosphere microenvironment. This improvement further upregulates key metabolic enzymes in shoots, accelerating carbohydrate synthesis and storage. However, some reports indicate that biological fertilizers universally increase nitrate reductase activity and reducing sugar content (Wang et al., 2025c). In contrast, these indicators did not increase significantly in our study. Furthermore, the T3 treatment even significantly decreased α-amylase activity. The specific sampling period directly caused this difference in physiological responses. At 90 days after transplanting, the tobacco leaves had entered the maturation and yellowing stage. The metabolic focus of the plant had shifted from vegetative growth to carbohydrate storage. This shift manifested as substantial starch accumulation and the suppression of starch-degrading α-amylase activity (Bonanomi et al., 2020). Concurrently, the demand for active nitrate reduction naturally weakened. Furthermore, differences in basic environmental conditions across study regions and the specific strain characteristics of the applied agents may also contribute to these stage-specific responses in physiological and metabolic indicators.

Rhizosphere microorganisms are central to driving soil nutrient cycling and maintaining plant health (Vejan et al., 2016). This study found that T4 and T2 significantly increased bacterial community richness (Chao1 and Ace indices). This increase occurred because the exogenous carbon sources and beneficial bacteria provided more abundant nutritional substrates for native microbes (Thepbandit and Athinuwat, 2024). Conversely, T3 significantly decreased community richness. This decrease indicates that this specific agent exerted a strong environmental screening or inhibitory effect on certain native microorganisms. Regarding community structure, different treatments exhibited distinctly different successional pathways. NMDS and genus-level analyses showed that the T4 treatment optimized the overall community structure. It achieved this optimization while maintaining the stable abundance of dominant native bacteria such as *Gp6* and *Nitrososphaera*. In contrast, the T3 treatment triggered a distinct shift in community structure. It substantially reduced the abundance of the aforementioned original dominant taxa and significantly enriched specific groups like *Tepidisphaera* and *Gp16*. Rather than relying solely on presumed microbial functions from the literature, our correlation analysis provides direct statistical support linking these microbial community shifts to plant performance. Specifically, bacterial α-diversity showed a highly significant negative correlation with plant potassium content and soil pH. The enrichment of these specific taxa aligns with their unique ecological functions in SOM turnover. Members of *Tepidisphaera* (phylum *Planctomycetota*) are known for their robust stress tolerance and ability to degrade complex biopolymers (English et al., 2025). This allows them to survive the initial environmental screening of the anti-continuous cropping agent and assist in degrading root exudate autotoxins. Concurrently, *Gp16* belongs to the phylum *Acidobacteriota*, which are widely known for decomposing recalcitrant carbon sources in soil (Shi et al., 2021). Therefore, the enrichment of these two groups helps break down complex organic matter and potential autotoxins. As verified by our multivariate analysis, this directional succession of the underlying rhizosphere microecological structure is statistically linked to the improved soil environment around the roots, directly facilitating the uptake and transfer of nutrients like phosphorus and potassium to the leaves during the late growth stage. These findings suggest that the T4 treatment tends to promote growth synergistically with native flora. Conversely, the T3 treatment improves the restricted microenvironment by disrupting the original community structure and promoting the proliferation of adaptable microbial populations. However, since this study lacks pre-transplanting baseline soil microbiome data, future research should incorporate initial soil sampling to dynamically track the successional pathways of these microbial communities over the entire growth period.

## 5 Conclusion

This study demonstrates that supplementing conventional organic fertilizers with functional agents can effectively overcome late-stage growth bottlenecks in flue-cured tobacco. Specifically, the addition of carbon polymer water-soluble fertilizer primarily provides early quick-acting nutrients. Building upon this foundation, the further addition of a microbial inoculant or an anti-continuous cropping agent exhibits significant late-stage growth-promoting advantages through distinct mechanisms. T4 maximizes late-stage dry matter accumulation by alleviating soil compaction (reducing BD), significantly increasing soil available nutrients, and synergistically activating leaf carbon and nitrogen metabolism. Conversely, T3 significantly alters soil acid-base conditions and drives the succession of the microbial community, enriching specific functional taxa to break down complex organic matter, thereby facilitating a unique nutrient allocation strategy. These distinct fertilization strategies can be flexibly applied in practical production based on specific soil conditions to optimize the rhizosphere environment. Given the limitations of this single-year, single-site field study, future multi-location field trials integrating multi-omics approaches and initial soil microbiome tracking are required to verify the stable efficacy of these combined strategies across broader ecological regions.

## Data Availability

The datasets presented in this study can be found in online repositories. The names of the repository/repositories and accession number(s) can be found below: https://www.ncbi.nlm.nih.gov/, PRJNA1443654.

## References

[B1] Ahmad M. Pataczek L. Hilger T. Zahir Z. A. Hussain A. Rasche F. . (2018). Perspectives of microbial inoculation for sustainable development and environmental management. Front. Microbiol. 9. doi: 10.3389/fmicb.2018.02992. PMID: 30568644 PMC6289982

[B2] Andrews M. Raven J. A. Lea P. J. Sprent J. I. (2005). A role for shoot protein in shoot–root dry matter allocation in higher plants. Ann. Bot. 97, 3–10. doi: 10.1093/aob/mcj009. PMID: 16299006 PMC2803373

[B3] Bao S. D. (2000). Soil and agricultural chemistry analysis (Beijing, China: Chinese Agriculture Press).

[B4] Bonanomi G. Alioto D. Minutolo M. Marra R. Cesarano G. Vinale F. (2020). Organic amendments modulate soil microbiota and reduce virus disease incidence in the TSWV-tomato pathosystem. Pathogens 9, 379. doi: 10.3390/pathogens9050379. PMID: 32423086 PMC7281679

[B5] Dutta P. Muthukrishnan G. Gopalasubramaiam S. K. Dharmaraj R. Karuppaiah A. Loganathan K. . (2022). Plant growth-promoting rhizobacteria (PGPR) and its mechanisms against plant diseases for sustainable agriculture and better productivity. Biocell 46, 1843–1859. doi: 10.32604/biocell.2022.019291

[B6] Eickholt D. P. Lewis R. S. (2014). Effect of an introgressed nicotiana tomentosa leaf number QTL on yield and quality characteristics in flue-cured tobacco. Crop Sci. 54, 586–594. doi: 10.2135/cropsci2013.07.0464

[B7] English C. J. Manoj M. Opalk K. Carlson C. A. (2025). Seasonal and developmental stage changes in exudate carbohydrate content shape the kelp microbiome. doi: 10.1101/2025.05.07.652744 PMC12642873 41293546

[B8] Gao J. Li L. Hu Z. Yue H. Zhang R. Xiong Z. (2015). Effect of ammonia stress on nitrogen metabolism of ceratophyllum demersum. Environ. Toxicol. Chem. 35, 205–211. doi: 10.1002/etc.3182. PMID: 26222052

[B9] Guo F. Wang S. Wang C. Wu S. Zhao X. Li G. (2023). N in granular compost accelerates crop use of soil N. doi: 10.21203/rs.3.rs-3301676/v1

[B10] Jalal A. Oliveira C. E. S. Galindo F. S. Rosa P. A. L. Gato I. M. B. Lima B. H. . (2023). Regulatory mechanisms of plant growth-promoting rhizobacteria and plant nutrition against abiotic stresses in brassicaceae family. Life. 13, 211. doi: 10.3390/life13010211. PMID: 36676160 PMC9860783

[B11] Jiang Y. Zhang R. Zhang C. Su J. Cong W.-F. Deng X. (2022). Long-term organic fertilizer additions elevate soil extracellular enzyme activities and tobacco quality in a tobacco-maize rotation. Front. Plant Sci. 13. doi: 10.3389/fpls.2022.973639. PMID: 36160995 PMC9501973

[B12] Lai X. Duan W. Zhang W. Peng Z. Wang X. Wang H. . (2024). Integrative analysis of microbiome and metabolome revealed the effect of microbial inoculant on microbial community diversity and function in rhizospheric soil under tobacco monoculture. Microbiol. Spectr. 12, e04046-23. doi: 10.1128/spectrum.04046-23. PMID: 38989997 PMC11302352

[B13] Lei B. Chang W. Zhao H. Zhang K. Yu J. Yu S. . (2022). Nitrogen application and differences in leaf number retained after topping affect the tobacco (nicotiana tabacum) transcriptome and metabolome. BMC Plant Biol. 22, 38. doi: 10.1186/s12870-022-03426-x. PMID: 35045826 PMC8767696

[B14] Li W. Zhang H. Li X. Zhang F. Liu C. Du Y. . (2017). Intergrative metabolomic and transcriptomic analyses unveil nutrient remobilization events in leaf senescence of tobacco. Sci. Rep. 7, 12126. doi: 10.1038/s41598-017-11615-0. PMID: 28935979 PMC5608745

[B15] Liang Y. Zhang M. Peng B. Zeng X. Li X. Wei J. (2025). Impact of fermented rapeseed cake mixed bacillus velezensis on the bacterial community structure and cultivation of tobacco cultivar K326. Sci. Rep. 15, 24818. doi: 10.1038/s41598-025-08400-9. PMID: 40640228 PMC12246417

[B16] Lin Y. DeJun K. Wang Z. Chen Y. Yang Z. Chun W. . (2020). Nitrogen application modifies the seed and oil yields and fatty acid composition of nicotiana tabacum. Hortscience 55, 1898–1902. doi: 10.21273/hortsci15335-20

[B17] Liu M. Liu X. Song Y. Hu Y. Yang C. Li J. . (2024). Tobacco production under global climate change: combined effects of heat and drought stress and coping strategies. Front. Plant Sci. 15. doi: 10.3389/fpls.2024.1489993. PMID: 39670262 PMC11635999

[B18] Malgioglio G. Rizzo G. F. Nigro S. Prey V. L. Herforth-Rahmé J. Catara V. . (2022). Plant-microbe interaction in sustainable agriculture: the factors that may influence the efficacy of PGPM application. Sustainability 14, 2253. doi: 10.3390/su14042253. PMID: 30654563

[B19] Mohamed M. H. M. Ali M. Eid R. S. M. El-Desouky H. S. Petropoulos S. A. Sami R. . (2021). Phosphorus and biofertilizer application effects on growth parameters, yield and chemical constituents of broccoli. Agronomy 11, 2210. doi: 10.3390/agronomy11112210. PMID: 30654563

[B20] Ouf S. A. El-Amriti F. A. Abu-Elghait M. Desouky S. E. Mohamed M. (2023). Role of plant growth promoting rhizobacteria in healthy and sustainable agriculture. Egypt. J. Bot. 63, 333–359. doi: 10.21608/ejbo.2023.191783.2246

[B21] Pavankumar S. Qadir J. Aryan S. Thakur R. Afreen S. (2024). An overview of biofertilizers in agriculture with special reference to mulberry. Int. J. Adv. Biochem. Res. 8, 389–397. doi: 10.33545/26174693.2024.v8.i5sf.1210

[B22] Saeed Q. Wang X. Haider F. U. Kučerík J. Mumtaz M. Z. Holátko J. . (2021). Rhizosphere bacteria in plant growth promotion, biocontrol, and bioremediation of contaminated sites: a comprehensive review of effects and mechanisms. Int. J. Mol. Sci. 22, 10529. doi: 10.3390/ijms221910529. PMID: 34638870 PMC8509026

[B23] Shi X. Zhao X. Ren J. Dong J. Zhang H. Dong Q. . (2021). Influence of peanut, sorghum, and soil salinity on microbial community composition in interspecific interaction zone. Front. Microbiol. 12. doi: 10.3389/fmicb.2021.678250. PMID: 34108953 PMC8180576

[B24] Smith M. R. Rao I. M. Merchant A. (2018). Source-sink relationships in crop plants and their influence on yield development and nutritional quality. Front. Plant Sci. 9. doi: 10.3389/fpls.2018.01889. PMID: 30619435 PMC6306447

[B25] Thepbandit W. Athinuwat D. (2024). Rhizosphere microorganisms supply availability of soil nutrients and induce plant defense. Microorganisms 12, 558. doi: 10.3390/microorganisms12030558. PMID: 38543610 PMC10975764

[B26] Trivedi P. Mattupalli C. Eversole K. Leach J. E. (2021). Enabling sustainable agriculture through understanding and enhancement of microbiomes. New Phytol. 230, 2129–2147. doi: 10.1111/nph.17319. PMID: 33657660

[B27] Vejan P. Abdullah R. Khadiran T. Ismail S. Boyce A. N. (2016). Role of plant growth promoting rhizobacteria in agricultural sustainability—a review. Molecules 21, 573. doi: 10.3390/molecules21050573. PMID: 27136521 PMC6273255

[B28] Wang C. Zheng M. Feng Y. Zhang S. Zhang Y. Liu F. . (2023). Effects of biochar-based fertilizer on soil physicochemical properties and rhizosphere bacterial community structure. Plant Nutr. Soil Sci. Int. 2, 1–19. doi: 10.23977/pnssi.2023.020101

[B29] Wang G. Ren Y. Bai X. Su Y. Han J. (2022). Contributions of beneficial microorganisms in soil remediation and quality improvement of medicinal plants. Plants 11, 3200. doi: 10.3390/plants11233200. PMID: 36501240 PMC9740990

[B30] Wang L. Feng Y. Chen Y. Zhang T. Zeng H. Zhang H. . (2025a). Synergistic bio-organic fertilization enhances tobacco antioxidative defense and soil health for sustainable agriculture. ACS Omega 10, 20001–20014. doi: 10.1021/acsomega.5c02029. PMID: 40415794 PMC12096250

[B31] Wang X. Huang J. Tan Y. Yang L. Li Y. Xia B. . (2025b). Synergistic effects of deep rotary tillage and microbial decomposition agents on straw decomposition, soil nutrient dynamics, and microbial communities in rice systems. Agriculture 15, 1447. doi: 10.3390/agriculture15131447. PMID: 30654563

[B32] Wang X. Sun M. Tian L. Yang M. Gao Q. Wang L. . (2025c). Microbial fertilizers modulate tobacco growth and development through reshaping soil microbiome and metabolome. Microbiol. Spectr. 13, e02605-24. doi: 10.1128/spectrum.02605-24. PMID: 40401958 PMC12211045

[B33] Yang X. Zhang K. Qi Z. Shaghaleh H. Gao C. Chang T. . (2024a). Field examinations on the application of novel biochar-based microbial fertilizer on degraded soils and growth response of flue-cured tobacco (nicotiana tabacum L.). Plants 13, 1328. doi: 10.3390/plants13101328. PMID: 38794400 PMC11125685

[B34] Yang Y. Ahmed W. Ye C. Yang L. Wu L. Dai Z. . (2024b). Exploring the effect of different application rates of biochar on the accumulation of nutrients and growth of flue-cured tobacco (Nicotiana tabacum). Front. Plant Sci. 15. doi: 10.3389/fpls.2024.1225031. PMID: 38463569 PMC10920355

[B35] Ying Z. Xing Y. Li Y. Jia J. Ying Y. Shi W. (2024). The role of phosphate-solubilizing microbial interactions in phosphorus activation and utilization in plant–soil systems: a review. Plants 13, 2686. doi: 10.3390/plants13192686. PMID: 39409556 PMC11478493

[B36] Yu L. Zhang M. Zhang S. Chen M. Yuan M. Huang J. . (2026). Root enhancement improves rhizosphere nutrient availability and promotes growth in flue-cured tobacco. Front. Plant Sci. 16. doi: 10.3389/fpls.2025.1728181. PMID: 41626324 PMC12854075

[B37] Zhang M. Guo D. Wang H. Wu G. Ding N. Shi Y. . (2024). Integrated characterization of filler tobacco leaves: HS–SPME–GC–MS, E-nose, and microbiome analysis across different origins. Bioresour. Bioprocess. 11, 11. doi: 10.1186/s40643-024-00728-w. PMID: 38647645 PMC10992047

